# Barriers to emergency department clinicians' confidence in providing paediatric trauma‐informed care

**DOI:** 10.1002/jcv2.12091

**Published:** 2022-07-21

**Authors:** Nimrah Afzal, Mark D. Lyttle, Eva Alisic, David Trickey, Rachel M. Hiller, Sarah L. Halligan

**Affiliations:** ^1^ Department of Psychology University of Bath Bath UK; ^2^ Emergency Department Bristol Royal Hospital for Children Bristol UK; ^3^ Research in Emergency Care Avon Collaborative Hub (REACH) University of the West of England Bristol UK; ^4^ Child and Community Wellbeing Unit Melbourne School of Population and Global Health University of Melbourne Melbourne Victoria Australia; ^5^ Anna Freud Centre London UK; ^6^ Faculty of Brain Science University College London London UK

**Keywords:** emergency department, paediatric injury, PTSD, trauma, traumatic stress

## Abstract

**Background:**

It has been estimated that around 31% of children will experience a traumatic event during childhood, most commonly serious accidents that lead to hospitalisation. Around 15% of children who experience such events go onto develop post‐traumatic stress disorder. Emergency department (ED) clinicians have a unique opportunity to intervene during the early peri‐trauma period, which can involve incorporating a trauma‐informed approach within their care. The available evidence indicates that clinicians internationally need further education and training to enhance their knowledge and confidence in providing trauma‐informed psychosocial care. However, UK/Ireland specific knowledge is limited.

**Methods:**

The current study analysed the UK and Irish subset of data (*N* = 434) that was collected as part of an international survey of ED clinicians. Questionnaires indexed clinician confidence in providing psychosocial care, and a range of potential barriers to providing that care. Hierarchical linear regression was used to identify predictors of clinician confidence.

**Results:**

Clinicians reported moderate levels of confidence in providing psychosocial care to injured children and families (*M* = 3.19, SD = 0.46). Regression analyses identified negative predictors of clinical confidence, including a lack of training, worrying about further upsetting children and parents, and low levels of perceived departmental performance in providing psychosocial care (*R* ^2^ = 0.389).

**Conclusions:**

The findings highlight the need for further training in psychosocial care for ED clinicians. Future research must identify nationally relevant pathways to implement training programmes for clinicians, in order to improve their skills in relation to paediatric traumatic stress and to reduce the perception of barriers identified in the present study.

**Key Points**

- Up to 40,000 children present to emergency departments (EDs) with acute injuries in the UK, and up to 16% of injured children develop post‐traumatic stress disorder
- ED can take a ‘trauma‐informed approach’ to care which facilitates both psychological and physical recovery following traumatic exposure. International research suggests that clinicians are not confident in providing trauma‐informed psychosocial care
- UK and Irish ED clinicians' confidence is related to a lack of training, worrying about further upsetting children and parents, and low levels of perceived departmental performance in providing psychosocial care
- These findings identify nationally relevant pathways to implementation of training for clinicians in paediatric traumatic stress

## INTRODUCTION

A third of children aged under 15 years visit UK emergency departments (EDs) annually (Keeble & Kossarova, 2017). Data suggest these rates are steadily rising, particularly amongst children under 5 years old (Ruzangi et al., 2020). Children and young people more frequently visit EDs than adults, with attendance rates at 425 for every 1000 children and 345 for every 1000 adults (NHS Digital, 2017). Common reasons for ED visitations include unintentional injuries, such as road traffic accidents, burns and animal bites (Public Health England, 2018). Such events can impact a child's mental health, including development of posttraumatic stress symptoms (PTSS), which can have substantial consequences for the wellbeing of the child and family (Bakker et al., 2013).

Symptoms of post‐traumatic stress disorder (PTSD) include re‐experiencing the traumatic event through intrusions, cognitive and behavioural avoidance of trauma material, hyperarousal, and negative alterations in cognitions and mood (American Psychiatric Association, 2013). Statistics suggest at least 88% of children and parents experience at least one acute posttraumatic stress symptom following ED attendance (Winston et al., 2003), and up to 16% of injured children develop clinically significant PTSD (Alisic et al., 2014; Winston et al., 2003). While 50% of initial cases are suggested to experience a decline in symptoms within 6 months without intervention, PTSD recovery beyond this 6‐month point is unlikely (Hiller et al., 2016). Consequently, post‐injury child PTSS requires clinical attention to prevent longer‐term negative sequelae. It is therefore important to consider intensity, timing and appropriateness of information and coping advice provided to children. ED clinicians are in a unique position to provide clinical guidance during this period and can identify acute PTSS (Alisic et al., 2014).

One method to support children's post‐trauma recovery is a ‘trauma‐informed approach’ (SAMHSA, 2014). This provides a framework for organisational awareness of traumatic stress following traumatic exposure (including acute injuries), focused on recognising signs and symptoms of post‐trauma mental health difficulties, and integrating understanding into practices and policies. This approach has four principles: (1) realising that the impact of trauma spreads from individuals to communities and across different settings; (2) recognising signs and symptoms of trauma; (3) responding in a manner grounded within an understanding of trauma; and (4) minimising re‐traumatisation (SAMHSA, 2014). Within paediatric EDs, this refers to clinicians recognising indicators of PTSS in injured children and parents and responding in a way which minimises negative emotional sequalae of traumatic experiences (Marsac et al., 2016).

Despite their potential role in supporting post‐trauma mental health, research suggests that ED clinicians do not feel confident in their knowledge of mental health assessment, diagnosis, and management (Jelinek et al., 2013; Weiland et al., 2011). In 2016, Alisic and colleagues conducted an international survey to assess ED staff's knowledge, attitudes, and confidence regarding traumatic stress in children and trauma‐informed psychosocial care. Findings suggested ED clinicians require greater training regarding child traumatic stress to improve their knowledge of PTSS, and confidence in recognising symptoms of acute stress, providing coping assistance for acute stress symptoms, and offering guidance on how and when to access mental health services (Alisic et al., 2016; Hoysted et al., 2017).

The applicability of international findings relating to trauma‐informed care for children to EDs in the UK and Ireland is currently unclear. The structure, organisation, training, and level of resourcing of EDs differs significantly between countries. This is especially relevant for the UK's NHS, a unique public health system compared to, for example, Australia and New Zealand's systems, which are a combination of public and private settings (Papanicolas & Smith, 2013). Different healthcare systems are likely to experience different pressures, such as the 4‐h target for NHS ED visitations, as well as variations in patient contact (Tynkkynen & Vrangbæk, 2018). Analysis of national factors influencing clinicians' confidence can help inform relevant, tailored reform options for the provision of trauma‐informed care in EDs.

We examined ED clinicians' confidence in providing paediatric trauma‐informed care within a UK and Ireland‐specific context, and explored predictors of this confidence using the UK and Irish data subset of the aforementioned international ED clinician survey (Alisic et al., 2016). We investigated the relationship between both department‐wide factors regarding psychosocial care and individual barriers experienced by UK and Irish ED clinicians, and their confidence levels. Specific aims were to examine: (i) UK and Irish ED clinicians' confidence in providing trauma‐informed psychosocial care to children and families, (ii) barriers experienced by ED clinicians in providing trauma‐informed care, (iii) whether ED clinical environments provide a positive context in terms of providing trauma‐informed care, and (iv) the extent to which barriers identified and clinical contextual factors contribute to clinician confidence in providing trauma‐informed psychosocial care to children.

## METHODS

### Data and participants

Data in the present study were collected as part of a larger international study (Alisic et al., 2016), which assessed ED clinicians' perspectives on traumatic stress and psychosocial care in children using a web‐based self‐report questionnaire. Participants in the original study were recruited through Paediatric Emergency Research Networks in North America, Europe and Australasia, national health care provider forums and associations, and a snowball approach to collect responses from countries with less professional organisation and associations.

The present study analysed the UK and Irish subset of the data, in a total of 434 participants from the UK and Ireland. These clinicians were recruited through Paediatric Emergency Research in the UK and Ireland (PERUKI; Lyttle et al., 2014).

### Measures

The original questionnaire comprised 65 items covering seven categories: demographic information, knowledge of traumatic stress, confidence in providing psychosocial care, barriers to providing psychosocial care, department's performance in providing psychosocial care, perspectives on training in psychosocial care and traumatic stress and further comments.

#### Barriers to providing psychosocial care

Barriers experienced by ED clinicians in providing paediatric trauma‐informed psychosocial care were assessed across six items: time constraints, lack of training, confusing evidence on what to do, worry about further upsetting children and families, lack of dedicated space to provide psychosocial care, and lack of support from supervisors. This measure was adapted from previous research with ED clinicians (Kassam‐Adams et al., 2015), and scored on a 3‐point Likert scale: 1 (not a barrier), 2 (somewhat a barrier), and 3 (significant barrier), recoded into binary variables for regression analysis: (0) barrier absent and (1) barrier present (defined as a score of 2 or 3 on the original scale).

#### Departmental support for psychosocial care

Departmental support for psychosocial care was assessed using 11 items which examined ED clinicians' perceptions of their department's performance in providing support for psychosocial care to children and families. Clinicians rated their ED's general performance in: provision of psychosocial care to injured children and their families, support for staff in managing their own emotional responses to patients' trauma, and use of scientific evidence for psychosocial care provision. Support for core elements of Psychosocial First Aid (The National Child Traumatic Stress Network, 2015) were measured: contact and engagement, safety and comfort, stabilisation, information gathering on current needs, practical assistance, connection with social support, information on coping, and linking with collaborative services (Shultz & Forbes, 2014). Each of the items were scored on a 4‐point Likert scale ranging from 1 (poor) to 4 (excellent).

Given the novelty of this measure of department support for psychosocial care, Principle Components Analysis (PCA) with direct oblimin rotation was conducted to determine whether meaningful subscales could be extracted for analysis for UK and Irish data. The Keiser‐Meyer‐Olkin measure indicated that sampling was adequate for the analysis (KMO = 0.91). Bartlett's Test of Sphericity (*χ* ^2^ (55) = 2604.51, *p* < 0.001) indicated correlations between variables were significantly different from zero. Two components explained the majority of the variance Eigenvalues (67.5%) and the scree plot suggested a two‐component model. Appendix S1 in the Supporting Information presents the component loadings after rotation.

Six items were retained in component 1, including ‘giving information on coping’, and ‘linking children/families with collaborative services’. Five items were retained in component 2, including ‘enhancing immediate and ongoing safety’ and ‘information gathering on current needs and concerns’. An examination of items clustering onto each component suggested that component 1 represents ED Support for Access to Psychosocial Care/Services component 2 represents ED Support for Immediate Stress Responses. Internal consistency was good across both components: component 1 (*α* = 0.88) and component 2 (*α* = 0.90).

#### Confidence in providing paediatric trauma‐informed care

Clinicians' confidence in providing trauma‐informed care to children and families was measured across 18 items, each scored on a 4‐point Likert scale ranging from 1 (not at all) to 4 (very). This measure was developed by Alisic et al. (2016) following previous research with ED clinicians (Alisic et al., 2014; Kassam‐Adams et al., 2015). The items captured key domains of psychosocial and physical care required for potentially trauma‐exposed children to prevent the development of emotional distress. Key domains of care included: recognising emotional distress, providing emotional assistance, eliciting trauma details, informing families about traumatic symptoms and how to access mental health support. An average confidence score ranging from 1–4 was used for the regression analysis in the present study (Alisic et al., 2016).

#### Covariates

Years of experience in patient care and profession were identified as covariates, as previous analyses have identified these as being significantly related to clinician confidence (Alisic et al., 2016). Years of experience was treated as a continuous variable whereas respondents' profession was coded as binary: (0) nurse and (1) physician.

### Analysis plan

Statistical analyses were performed using IBM SPSS Statistics 27 (IBM Corp, 2017). Descriptive statistics were computed to explore respondent characteristics, confidence, barriers to implementing psychosocial care and departmental support for psychosocial care. Bivariate correlations were computed to assess whether relationships between the main variables were statistically significant (*p* < 0.05) (Appendix S2). Variables that were statistically significant were included in the regression analysis. The second component from the PCA analysis (ED support for immediate stress responses) was reversed as the items were inversely loaded onto the component; reversing therefore ensured the correct direction in the regression analysis. A hierarchical linear regression analysis was conducted to investigate predictors of clinicians' confidence in providing paediatric psychosocial care. Variables were entered into the model using a stepwise strategy: (1) clinicians' years of experience and profession, (2) the two department support scales (capturing departmental support for (a) access to psychosocial care/services, and (b) immediate stress responses), and (3) barriers to providing psychosocial care. The final model retained variables that were significantly related to clinician confidence.

## RESULTS

Characteristics of survey respondents from the UK (90.8%) and Ireland (9.2%) (*N* = 434) are outlined in Table 1. Respondents most frequently reported their department was a designated trauma centre (61.8%, *N* = 268), they worked in paediatric ED (64.1%, *N* = 278), and no previous training in psychosocial care (81.1%, *N* = 352).

**TABLE 1:** Characteristics of UK and Irish survey respondents

	*M* (SD)	*N*
Age	38.2 (9.01)	434
Years of experience in patient care	8.81 (7.00)	434

	*N* (%)	*N*
Gender		434
Male	131 (30.2)	
Female	303 (69.8)	
Profession		415
Nurse	198 (47.7)	
Physician	217 (52.3)	
Department		434
Paediatric ED	278 (64.1)	
Combined paediatric and adult ED	118 (27.2)	
Adult ED where children (sometimes) present	24 (5.5)	
Adult ED where children never present	2 (0.5)	
Other	12 (2.8)	
Location		434
Rural	14 (3.2)	
Suburban	61 (14.1)	
Urban	359 (82.7)	
Previous training in psychosocial care		388
Yes	36 (9.3)	
No	353 (90.7)	
Desire for further training in psychosocial care		389
Yes	358 (92.0)	
No	31 (8.0)	

Abbreviation: ED, emergency department.

### Confidence in providing trauma‐informed care

Table 2 outlines mean scores for individual clinician confidence items in descending order. The mean overall confidence score in providing trauma‐informed care amongst the survey respondents was 3.19 (SD = 0.46), which indicates a moderate level of confidence (a score of 3 = moderately confident). The top three items which clinicians reported being ‘very’ confident in were: (i) explaining medical procedures to children and parents, (ii) talking with children in age‐appropriate language, and (iii) assessing and managing pain in children. Conversely, items where most respondents reported being only ‘a little’ confident were: (i) providing information to parents about emotional/behavioural reactions that indicated the child may need help, and (ii) educating children and families about common traumatic stress reactions.

**TABLE 2:** Respondents' confidence in providing psychosocial care

How confident are you that you can…	Not at all (%)	A little (%)	Moderately (%)	Very (%)	Mean score (SD)
Explain procedures to children and parents	0.5	2.2	14.4	82.9	3.80 (0.49)
Talk with children in age‐appropriate language	0.2	3.2	15.3	81.2	3.78 (0.50)
Assess and manage pain in children	0.7	2.0	23.2	74.1	3.71 (0.54)
Take action to get someone close (a parent, family member or friend) available to the child in the ED	0.2	3.5	24.9	71.4	3.67 (0.55)
Respond calmly and without judgement to a child's or family's strong emotional distress	0.5	3.4	33.9	62.2	3.58 (0.59)
Liaise with staff who can provide practical assistance to a family (e.g., social work)	0	7.7	32.4	59.9	3.52 (0.64)
Encourage parents to make use of their own social support system (family, friends, spiritual community, etc.)	0.7	10.7	39.8	48.8	3.37 (0.70)
Manage your own emotional responses to children's pain and trauma	1.2	8.7	43.0	47.0	3.36 (0.69)
Tailor your approach according to a family's cultural background	1.2	7.8	52.9	38.0	3.28 (0.66)
Assess a child's or family's distress, emotional needs, and support systems	1.7	15.1	55.6	27.7	3.09 (0.70)
Respond to a child's (or parent's) question about whether the child will die	4.5	17.8	48.5	29.2	3.02 (0.81)
Teach parents or children specific ways to cope with procedures in the ED	2.5	23.0	46.0	28.5	3.01 (0.79)
Elicit trauma details from a child or family without them being exposed to more distress	5.4	19.0	49.6	25.9	2.96 (0.82)
Help a child/parent who is anxious to calm down by teaching relaxation (e.g., breathing) techniques	6.4	24.3	41.8	27.5	2.90 (0.88)
Inform a child about an injured/deceased family member	9.3	19.9	43.7	27.0	2.88 (0.91)
Educate parents or children about how to access mental health services if needed	7.2	34.7	41.7	16.4	2.67 (0.83)
Provide information to parents about emotional or behavioural reactions that indicate that the child may need help (when back at home)	14.4	40.8	33.6	11.2	2.42 (0.87)
Educate children and families about common traumatic stress reactions	14.2	41.4	35.4	9.0	2.39 (0.84)

*Note*: *N* = 379.

Abbreviation: ED, emergency department.

### Barriers to trauma‐informed care

**FIGURE 1:**
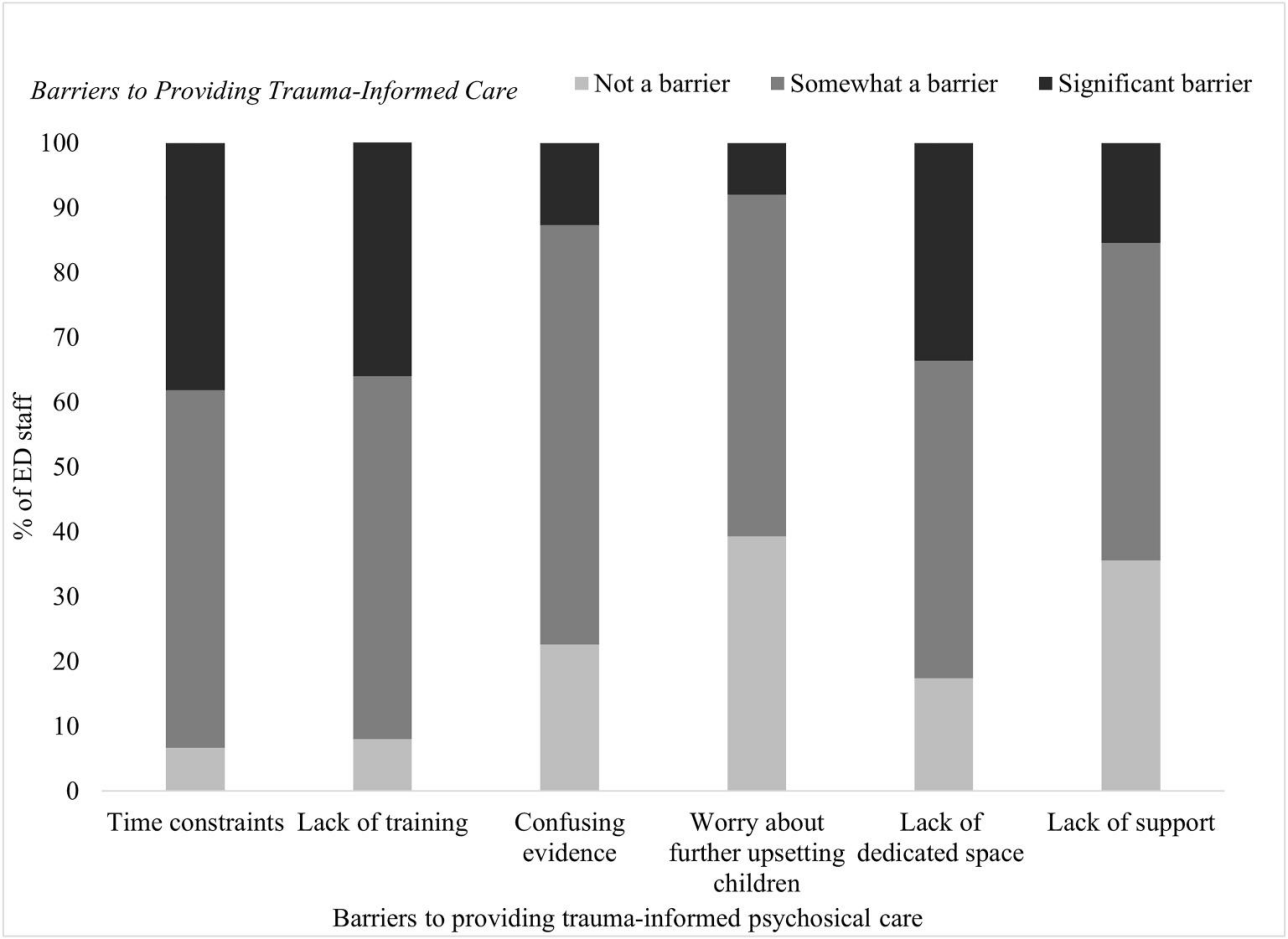
Percentages indicate the extent to which specific barriers to providing trauma‐informed care are experienced by ED clinicians. ED, emergency department

Barriers to providing trauma‐informed care are illustrated in Figure 1 (*N* = 402). Across all six barriers the majority of the sample endorsed them as being present (rated as either somewhat or significant barrier). The barriers most frequently identified as significant were time constraints (38.1%), lack of training (36.1%) and lack of dedicated space for psychosocial care (33.6%).

### Departmental performance in trauma‐informed care

Table 3 outlines respondents' perspectives on departmental performance in trauma‐informed care in descending order according to average departmental performance. Across the 11 items, the mean perceived departmental performance in psychosocial care was 2.67 (SD = 0.59), indicating that the average rating of ED performance in providing psychosocial care to injured children and families was ‘fair’. Mean perceived department performance in supporting access to psychosocial care was significantly lower (*M* = 2.43, SD = 0.66) than the mean perception of department performance in supporting immediate stress responses (*M* = 2.97, SD = 0.62), *t* (383) = −20.4, *p* < 0.001. However, across the two components, only a minority perceived their department to be performing ‘excellently’ in various aspects of psychosocial care, indicating opportunity for improvement and change.

**TABLE 3:** Departmental support for psychosocial care

Rate the performance of your ED in…	Poor (%)	Fair (%)	Good (%)	Excellent (%)	Mean score (SD)
Component 1: Departmental support for access to psychosocial care/services					
Connecting children/families with social supports	7.3	31.6	44.1	17.0	2.71 (0.83)
Helping staff manage their own emotional responses to patients' pain and trauma	9.1	36.1	44.2	10.6	2.56 (0.80)
Providing psychosocial care to injured children & families	9.7	35.2	45.4	9.7	2.55 (0.80)
Linking children/families with collaborative services	13.1	37.9	38.6	10.4	2.46 (0.85)
Giving information on coping	18.5	44.1	30.5	6.8	2.26 (0.84)
Using scientific evidence as basis for psychosocial care for patients & staff	31.1	41.3	24.2	3.4	2.00 (0.83)
Overall mean					2.43 (0.66)

Component 2: Departmental support for immediate stress responses					
Stabilisation (calming and orienting emotionally overwhelmed/distraught children/families)	1.3	16.4	56.1	26.1	3.07 (0.69)
Safety and comfort (enhancing immediate and ongoing safety, and provide physical and emotional comfort)	2.6	17.2	54.0	26.1	3.04 (0.73)
Contact and engagement (responding to or initiating contacts in a non‐intrusive, compassionate, and helpful manner)	3.4	18.8	54.3	23.5	2.98 (0.75)
Information gathering on current needs & concerns	3.1	22.5	52.2	22.2	2.93 (0.75)
Practical assistance (offering practical help to children/families in addressing immediate needs and concerns)	5.2	26.8	49.6	18.4	2.81 (0.79)
Overall mean					2.97 (0.62)

*Note*: *N* = 385.

Abbreviation: ED, emergency department.

### Associations with clinician confidence

Table 4 outlines variables that were retained in the regression model as they were significantly related to clinician confidence. In the final model lower clinician confidence was negatively related to two barriers: (i) worrying about further upsetting children (*β* = −0.222), and (ii) lack of training (*β* = −0.150). In addition, sum scores from scales measuring ED support for access to psychosocial care/services (*β* = 0.271) and support for immediate stress responses (*β* = 0.145) were positively associated with clinician confidence. Model co‐variates were also significant, with greater years of experience and being a nurse (vs. physician) predicting higher confidence scores. In the resultant model these variables explained 39% of the variance in clinicians' confidence in providing paediatric psychosocial care, (*F* = 28.00, *df *= 8, 352, *p* < 0.001), *R* ^2^ = 0.389.

**TABLE 4:** Predictors of ED clinicians' confidence in providing paediatric trauma‐informed care

Variables	*B* (SE)	*β*	95% CI
Years of experience in patient care	0.012 (0.002)	0.223***	0.007, 0.016
Profession^a^	−0.158 (0.039)	−0.175***	−0.235, −0.081
ED support for access to psychosocial care/services^b^	0.123 (0.023)	0.271***	0.077, 0.169
ED support for immediate psychosocial care^b^	0.066 (0.024)	0.145**	0.019, 0.113
Worry about further upsetting children^c^	−0.204 (0.043)	−0.222***	−0.289, −0.120
Lack of training^c^	−0.258 (0.077)	−0.150***	−0.410, −0.106

*Note*: *N* = 361.

Abbreviation: ED, emergency department.

^a^ Profession coded 0 = nurse, 1 = physician.

^b^ ED support scales.

^c^ Barriers to implementing trauma‐informed care.

***p ≤ 0.001; **p < 0.01; *p < 0.05.

## DISCUSSION

Overall, these findings suggest that within the UK and Ireland, specific barriers and aspects of the clinical environment within EDs contribute to clinician confidence in providing trauma‐informed psychosocial care to families and trauma‐exposed children. Specifically, worrying about further upsetting children, a lack of training and reduced ED support for providing psychosocial care were related to reduced clinician confidence. These findings contribute to the existing evidence base which highlights the need for further training in psychosocial care for ED clinicians globally (Alisic et al., 2016; Hoysted et al., 2017; Marsac et al., 2016; Weliand et al., 2011). However, they also provide an insight into barriers specific to the UK and Ireland. Previous analyses conducted on the original dataset have explored clinician confidence across international contexts, and the barriers that limit clinician confidence have not been systematically examined (Alisic et al., 2016; Hoysted et al., 2017). Given global variation in healthcare systems (Dijkink et al., 2017), and in particular the public nature of the UK National Health Service, extrapolation of previous findings to the UK and Ireland is potentially problematic. Therefore, the present study provides an understanding of which barriers are specifically related to clinician confidence of UK and Irish ED Clinicians. This can guide the identification of nationally‐specific pathways for implementation of training programmes in trauma‐informed care.

The current analysis found that rates of UK and Irish clinician confidence were highest amongst aspects of trauma‐informed care based on medical knowledge, including explaining medical procedures to children/parents and assessing and managing pain in children. Importantly, rates of confidence were lower for areas which required clinicians to use psychosocial knowledge, including providing information about traumatic symptoms, accessing relevant mental health services and teaching relaxation techniques (e.g., breathing). These findings are consistent with previous international research, in which ED clinicians reported lower rates of confidence in domains of trauma‐informed care requiring specific post‐traumatic stress (PTS) knowledge, as opposed to domains of pain management (Moss, Ziviani et al., 2019). The current results are also consistent with previous findings from the Australian/New Zealand and international scores in the wider dataset. Specifically, the findings are consistent with reports of clinician confidence being higher for physical/medical support versus psychological/social support (Alisic et al., 2016; Hoysted et al., 2017).

Lack of clinician confidence in providing psychosocial care for trauma exposed children is an important issue. For children and families exposed to trauma, time in the ED can be a confusing or frightening experience, and parents have expressed concerns about the lack of emotional support and posttrauma recovery guidance (physical and emotional) from clinicians, despite presentations of acute distress (Williamson et al., 2016). Importantly, severity of acute distress posttrauma in injured children and parents can be predictive of later PTSD (Kassam‐Adams & Winston, 2004; Kassam‐Adams et al., 2008). Thus, limiting child and parental distress and actively preventing re‐traumatisation may have longer term consequences for wellbeing (Marsac et al., 2016). Therefore, presentation at the ED may be a key clinical contact point at which children displaying disabling acute stress responses could be identified and appropriate signposting to future resources provided (Hiller et al., 2016).

Clinicians in the current sample identified significant obstacles to providing such psychosocial care. Notably, a high proportion perceived time constraints and lack of training as barriers. Previous research has highlighted that time constraints are a prominent obstacle in providing psychosocial care, as medical needs are prioritised over psychological needs within ED time restrictions (Alisic et al., 2014; Hoysted et al., 2017; Moss, Healy et al., 2019). However, the present analyses also identified which barriers were robustly and independently related to clinician confidence and identified that clinician confidence was influenced by a lack of training and worrying about further upsetting children. Dueweke et al. (2019) found that brief training in psychosocial care not only increased clinician confidence, but also reduced their perception that they did not have time to make this provision. Therefore, whilst interventions must be sensitive to space and time constraints, it is also possible that perceptions of these barriers are a consequence of limited knowledge of what providing psychosocial care entails.

The present analyses also suggest wider departmental culture is related to clinician confidence in trauma‐informed care. The contribution of departmental culture to clinician confidence has not been investigated by previous analyses conducted on the wider international dataset (Alisic et al., 2016). The current analysis suggests that poorer perceived departmental performance in providing immediate psychosocial care and informing families about how to access psychosocial care were both related to reduced clinician confidence in the UK and Ireland. These findings support suggestions that a department‐wide culture which recognises the importance of trauma‐informed care, and readily implements measures to facilitate this, is vital to providing effective support for trauma‐exposed children and their families (Marsac et al., 2016). More generally there is evidence that the organisational environment within EDs shapes clinicians' own attitudes and confidence when addressing mental health difficulties in patients (Clarke et al., 2014). Therefore, a department‐wide commitment to improving psychosocial aspects of trauma‐informed care is likely to be particularly effective in supporting clinicians in integrating this into their interactions with trauma‐exposed children and their families (DeCandia et al., 2014).

Overall, the current analyses highlight a clear need to improve UK and Irish ED clinicians' understanding and application of PTS‐specific knowledge. Previous research highlights that a lack of relevant training leads to inconsistencies in clinicians' knowledge of PTS following paediatric injury, and a reliance on providing psychosocial care based upon experience, rather than skills acquired through training (Alisic et al., 2014; Hoysted et al., 2017). Therefore, training is needed to equip clinicians with the necessary skills to provide emotional support and to offer anticipatory guidance for trauma recovery and service access.

Previous studies have investigated brief, trauma‐informed training programmes which aimed to improve clinicians' knowledge of traumatic stress and ability to translate knowledge into practice. Hoysted et al. (2019) conducted a pilot trial of a brief, low cost, web‐based training programme in Australia and New Zealand and found improvements in ED staff's knowledge in traumatic stress. Similarly, Dueweke et al. (2019) conducted a feasibility trial of a 2‐h training curriculum addressing paediatric traumatic stress in primary care. This involved teaching paediatric residents about trauma, a brief screening tool for PTS and how to carry out referrals. Preliminary results included increased perceived competence to carry out psychosocial trauma‐informed care, as well as decreases in perceiving time constraints and lack of training as barriers to providing psychosocial care. These results suggest that it is feasible to integrate brief clinician training in paediatric traumatic stress to improve confidence in providing psychosocial care and decrease the perception that clinicians do not have time to provide such care. However, the effectiveness of such programmes on clinician performance and long‐term confidence has not been tested. Moreover, training programmes have not been examined within the UK and Ireland. Therefore, future research must identify UK and Irish‐specific pathways to the implementation of training programmes that address the barriers identified within the current study.

Whilst the present study provides a valuable insight into barriers related to UK and Irish ED clinician confidence, the cross‐sectional nature of the data cannot support any conclusions regarding the direction of the effects. Therefore, it is not possible to conclude that the barriers cause reduced clinician confidence. Moreover, the use of self‐reporting may have also impacted respondents' answers and as such, they may not have honestly responded regarding their own confidence and their departments' competence in providing psychosocial care.

A further limitation is sample representativeness. The participants for this analysis were recruited via Paediatric Emergency Research in the UK and Ireland (Lyttle et al., 2014). Therefore, respondents may have already been interested in paediatric trauma‐unformed care, which could have biased the results as sites with less interest in, or poorer clinician confidence/performance in trauma‐informed care, may not have been represented.

Future research must also consider the impact of the COVID‐19 pandemic which has significantly impacted the NHS with increased numbers of ED attendances (Panovska‐Griffiths et al., 2021). The data used in the present study was collected prior to the pandemic and therefore, it is not possible to determine how the pandemic has impacted clinicians' perception of these barriers. Therefore, future research can benefit from updating these findings, and utilising longitudinal study designs and recruitment from a wider pool of paediatric EDs.

## CONCLUSION

The present study highlights that UK and Irish ED clinicians' confidence in providing paediatric trauma‐informed psychosocial care is related to several barriers. These findings points towards a need to improve (a) individually experienced barriers by clinicians, and (b) facilitating a department‐wide trauma‐informed culture. A potential solution is to offer brief training programmes to ED clinical staff to educate them in recognising symptoms of acute distress, triaging children for further support and reducing immediate symptoms of acute distress in children and parents. Previous training curriculums have shown promising results in reducing clinician's perception of barriers and increasing clinician confidence. However, future research must identify pathways relevant to UK and Irish EDs to implement training programmes that aim to reduce the perception of barriers identified in the present study.

## AUTHOR CONTRIBUTIONS

**Nimrah Afzal**: Conceptualization; Formal analysis; Investigation; Methodology; Writing – original draft. **Mark D. Lyttle**: Data curation; Supervision; Writing – review & editing. **Eva Alisic**: Data curation; Investigation; Resources; Validation; Writing – review & editing. **David Trickey**: Writing – review & editing. **Rachel M. Hiller**: Supervision; Writing – review & editing. **Sarah L. Halligan**: Conceptualization; Supervision; Writing – review & editing.

## CONFLICT OF INTEREST

The authors have declared that they have no competing or potential conflicts of interest.

## ETHICAL CONSIDERATIONS

The original data collection was approved by the Human Research Ethics Committee of the Royal Children's Hospital Melbourne (HREC 33085).

## Supporting information

Supporting Information S1

Supporting Information S2

## Data Availability

An anonymised dataset is available from Eva Alisic, PhD, University of Melbourne. Email: ealisic@unimelb.edu.au.
