# Supplementary material for: Barriers to emergency department clinicians' confidence in providing paediatric trauma‐informed care
Source: JCPP Adv. 2022 Jul 21;2(3):e12091. doi: 10.1002/jcv2.12091 (PMC10242876; doi:10.1002/jcv2.12091)
Supplement: Supplementary file 1 — Supporting Information S1 [file JCV2-2-e12091-s001.docx]

| **Supporting Information - Appendix S1**  *Principle Components Analysis Loadings of Items Measuring Department Performance* | | |
| --- | --- | --- |
| **Item** | **Component 1** | **Component 2** |
| Providing psychosocial care to injured children & families | .65 |  |
| Helping staff manage their own emotional responses to patients’ pain and trauma | .59 |  |
| Using scientific evidence as basis for psychosocial care for patients & staff | .89 |  |
| Contact and engagement  (responding to or initiating contacts in a non-intrusive, compassionate, and helpful manner) |  | -.83 |
| Safety and comfort (enhancing immediate and ongoing safety, and provide physical and emotional comfort) |  | -.92 |
| Stabilisation (calming and orienting emotionally overwhelmed/distraught children/families) |  | -.93 |
| Information gathering on current needs & concerns |  | -.68 |
| Practical assistance  (offering practical help to children/families in addressing immediate needs and concerns) |  | -.54 |
| Connecting children/families with social supports | .67 |  |
| Giving information on coping | .86 |  |
| Linking children/families with collaborative services | .87 |  |
| *Note.* PCA loadings of less than .30 were suppressed |  |  |
