# Supplementary material for: Barriers to emergency department clinicians' confidence in providing paediatric trauma‐informed care
Source: JCPP Adv. 2022 Jul 21;2(3):e12091. doi: 10.1002/jcv2.12091 (PMC10242876; doi:10.1002/jcv2.12091)
Supplement: Supplementary file 2 — Supporting Information S2 [file JCV2-2-e12091-s002.docx]

|  | | | | | | | | | |
| --- | --- | --- | --- | --- | --- | --- | --- | --- | --- |
| **Supporting Information – Appendix S2**  *Correlations Between Main Study Variables* | | | | | | | | | |
|  | ED support for access to social care/services | ED support for immediate stress responses | Average clinician confidence in trauma-informed care | Time constraints^a^ | Lack of training^a^ | Confusing evidence on what to do^a^ | Worry about further upsetting children and families^a^ | Lack of a dedicated space to provide psychosocial care^a^ | Lack of support from supervisors^a^ |
| ED support for access to social care/services | 1 |  |  |  |  |  |  |  |  |
| ED support for immediate stress responses | -.584^***^ | 1 |  |  |  |  |  |  |  |
| Average clinician confidence in trauma-informed care | .406^***^ | .368^***^ | 1 |  |  |  |  |  |  |
| Time constraints^a^ | -.078 | -.047 | -.025 | 1 |  |  |  |  |  |
| Lack of training^a^ | -.196^***^ | -.163^**^ | -.286^***^ | .068 | 1 |  |  |  |  |
| Confusing evidence on what to do^a^ | -.175^**^ | -.168^***^ | -.104^*^ | .069 | .346^***^ | 1 |  |  |  |
| Worry about further upsetting children and families^a^ | -.122^*^ | -.170^***^ | -.309^***^ | .130^**^ | .215*^**^ | .307*^**^ | 1 |  |  |
| Lack of a dedicated space to provide^a^ psychosocial care | -.171^***^ | -.221^***^ | -.153^**^ | .270^***^ | .132^**^ | .175^***^ | .275^***^ | 1 |  |
| Lack of support from supervisors^a^ | -.171^***^ | -.203^***^ | -.046 | .195^***^ | .185^***^ | .318^***^ | .296^***^ | .358^***^ | 1 |
| *Note.* *N* = 375.  ^a^Barriers to implementing trauma-informed care.  ****p* ≤ .001. ***p* < .01. **p* < .05. | | | | | | | | | |
